# Supporting Data for Multifunctional all-in-one drug delivery systems for tumor targeting and sequential release of three different anti-tumor drugs

**DOI:** 10.1016/j.dib.2016.02.026

**Published:** 2016-02-15

**Authors:** Guowei Wu, Chaojun Song, Renata Grifantini, Li Fan, Hong Wu, Boquan Jin

**Affiliations:** a Department of Pharmaceutical Analysis, The Fourth Military Medical University, Xi’an, Shaanxi 710032, China; b Department of Immunology, The Fourth Military Medical University, Xi’an, Shaanxi 710032, China; c Externautics SpA, Siena, SI 53100, Italy; d Department of Physics, Chinese University of Hong Kong, Shatin, Hong Kong

## Abstract

Although nanoparticulate drug delivery systems (NDDSs) can preferentially accumulate in tumors, active targeting by targeting ligands (e.g. monoclonal antibody) is necessary for increasing its targeting efficacy in vivo. We conjugated mAb198.3 on the SiO_2_@AuNP system surface to make it obtain active targeting efficacy. The FAT1 targeting capability of SiO_2_@AuNP system is the first issue to be solved. Thus, flow cytometry analysis was attempted to demonstrate that the SiO_2_@AuNP system could bind to native FAT1 molecules on the surface of Colo205 cells. Also, together with the drug release behavior study of self-decomposable SiO_2_ NPs, the continuous morphological evolution needed to be clarified. Therefore, to characterize the morphological evolution in vitro, we analyzed the morphology of inner self-decomposable NPs in different time intervals using transmission electron microscopy (TEM). A more comprehensive analysis of this data may be obtained from the article “Multifunctional all-in-one drug delivery systems for tumor targeting and sequential release of three different anti-tumor drugs” in Biomaterials.

**Specifications Table**

**Table:** 

Subject area	Immunology, Chemistry, Biology
More specific subject area	Targeting and drug release
Type of data	Figures
How data was acquired	Flow cytometry and transmission electron microscopy analysis
Data format	Normalized data
Experimental factors	Targeting efficacy and morphology of NP during drug release process
Experimental features	The in vitro targeting efficiency of the SiO_2_@AuNP system was determined by flow cytometry analysis in vitro. Also, The degradation of the SiO_2_ carrier was monitored by a morphology investigation using TEM.
Data source location	The Fourth Military Medical University, Xi’an, Shaanxi, China
Data accessibility	The data is with this article and as supporting information to paper published in Biomaterials, “Multifunctional all-in-one drug delivery systems for tumor targeting and sequential release of three different anti-tumor drugs”.

**Value of the data**

• SiO_2_@AuNP system with high FAT1 targeting efficacy could be developed for multidrug sequential delivery in colon cancers.
• The approach we took to analysis of FAT1 targeting efficacy in SiO_2_@AuNP system using FACS could be useful to others.
• The continuous morphological evolution behavior of the inner self-decomposable SiO_2_ NPs monitored by TEM could be useful to others as providing another evidence of drug release.

## **1. Data, experimental design, materials and methods**

FAT1 targeting efficacy of SiO_2_@AuNP system was evaluated using FACS assay. The continuous morphological evolution behavior of the inner self-decomposable SiO2 NPs was monitored by TEM.

After demonstrating the targeting capability of free mAb198.3_Cy5 and Au-PEG-(Cy5)_mAb198.3 in our previous paper [1], we further double attached mAb198.3 and siRNA onto AuNP system. The obtained Au-PEG-198.3/siRNA NP was then absorbed on the surface of self-decomposable NP by electrostatic force. The in vitro targeting efficiency against FAT1 molecules of the SiO_2_@AuNP system was determined by flow cytometry analysis in vitro.

Reaction of the SiO_2_@AuNP with Colo205 cells was analyzed using flow cytometry. Colo 205 cells were harvested and incubated with SiO_2_@AuNP (40 μg/ml) for 30 min at 4 °C. After incubation, cells were washed for three times and resuspended in PBS. Samples were analyzed using a FACS Calibur flow cytometer and CellQuest™ Pro software (BD Bioscience, San Jose, CA). Each experiment was done in triplicate.

**Fig. 1:**
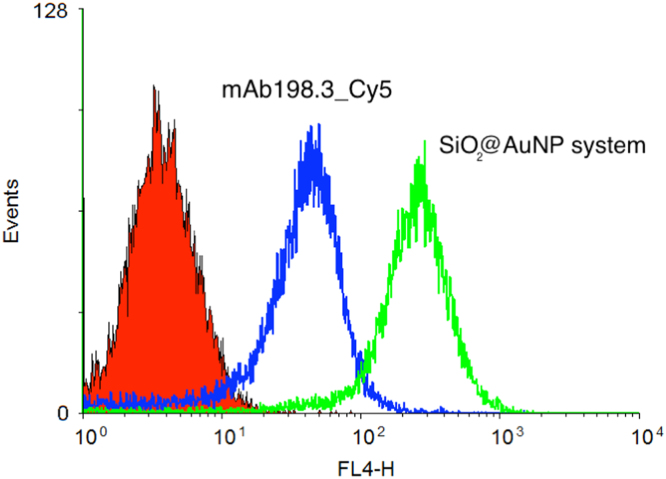
Flow cytometry analysis investigated the binding capability of SiO_2_@AuNP system to native FAT1 molecules on the surface of Colo205 cells.

Flow cytometry analysis demonstrated that the SiO_2_@AuNP system could bind to native FAT1 molecules on the surface of Colo205 cells (Fig. 1), and this result was consistent with our previous work on free mAb198.3_Cy5 and Au-PEG-(Cy5) _mAb198.3. This indicated that the SiO_2_@AuNP system has the FAT1 targeting capability (Fig. 1).

After binding to membranes, SiO_2_@AuNP started its endocytosis process, then endosome/lysosome escape to cytoplasm. The drug release behavior in the cytoplasm of the SiO_2_@AuNP system has been investigated in our submitted paper entitled “Multifunctional all-in-one drug delivery systems for tumor targeting and sequential release of three different anti-tumor drugs”. Together with the drug release, the continuous morphological evolution of the inner self-decomposable SiO_2_ NPs was also observed using TEM. Equal amounts of SiO_2_-HCPT/Dox NPs (1 mg/mL) were dispersed in 10 mL deionized water and centrifuged (10,000 rpm for 5 min) at a different time point. The precipitate of each sample was re-dispersed in deionized water. Transmission electron microscopy was done to intuitive study the morphology of particle on a JEOL 7C device at 120 kV. Samples were prepared by adding a drop (2–3 μL) of re-dispersed NP solution on a copper grid carrying a 20 nm thick carbon film (CF-300-Cu, Electron Microscopy Sciences), and drying for 1 h.

**Fig. 2:**
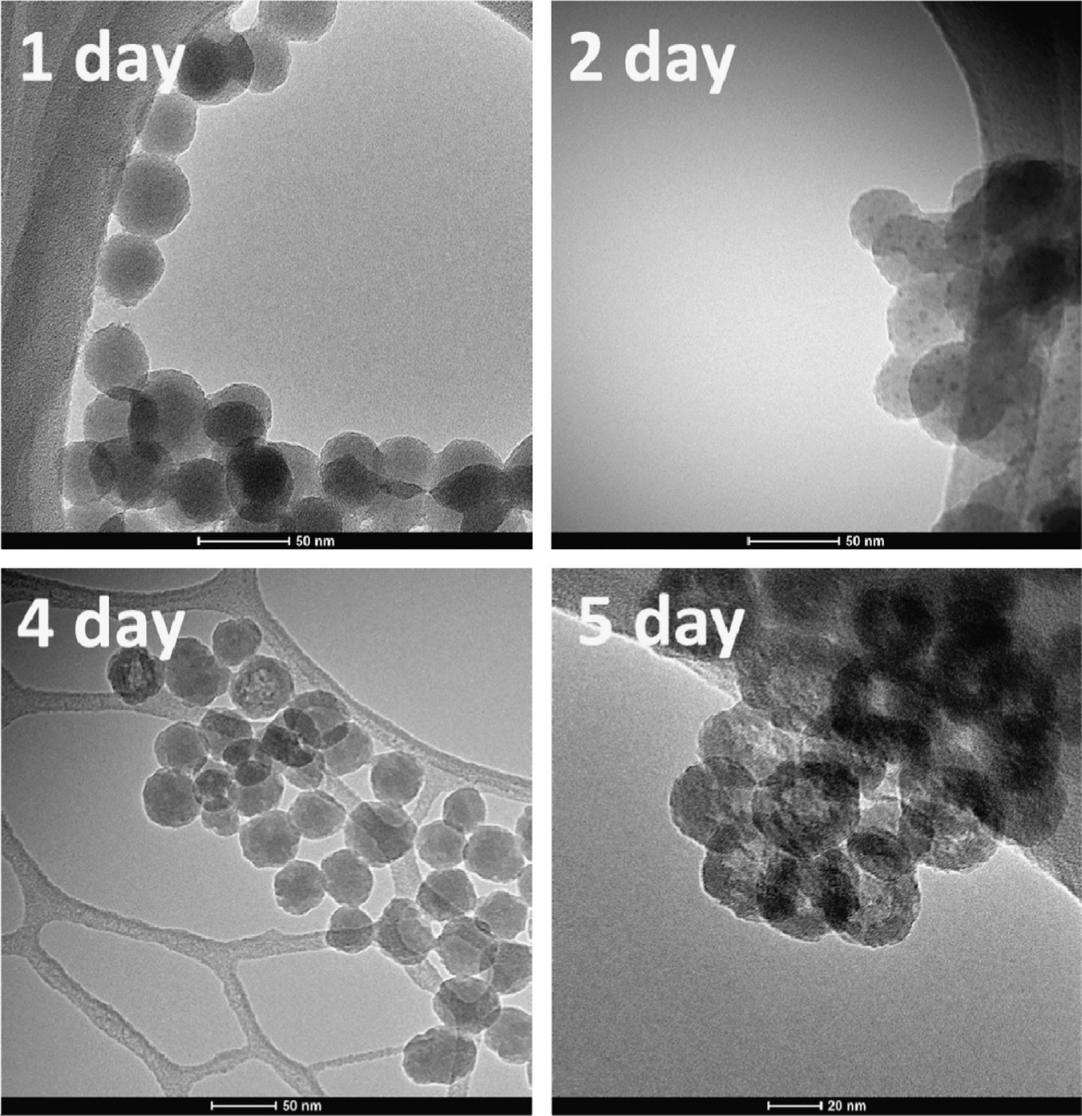
TEM images of NPs after being immersed in deionized water at 37 °C for different time intervals.

The results showed that most of the NPs remained intact at the first 4 h, while rough edges appeared after 6 h of immersion in deionized water. With the elongation of the immersed hours, obvious hollow features gradually appeared in the center of the NPs. Such uniform center-hollow features continued to enlarge over 48 h, leaving a spherical, thin, discontinuous shell (Fig. 2).
